# Conversion Paralysis After Cervical Surgery: A Case Report and Literature Review

**DOI:** 10.3389/fsurg.2022.814498

**Published:** 2022-02-17

**Authors:** Miao Fang, Jiaojiao Zhou, Yong Zeng, Shishu Huang, Yueming Song

**Affiliations:** ^1^Department of Orthopedics, Chengdu Second People's Hospital, Chengdu, China; ^2^Department of Orthopedics, West China Hospital of Sichuan University, Chengdu, China; ^3^Division of Ultrasound, West China Hospital of Sichuan University, Chengdu, China

**Keywords:** conversion paralysis, paralysis, cervical spondylotic myelopathy, cervical surgery, ACCF

## Abstract

We report a case of conversion paralysis triggered by cervical surgery that was caused by cervical spondylotic myelopathy (CSM). A 67-year-old man underwent anterior cervical corpectomy decompression and fusion for CSM. Upon awakening from the anesthesia, he had incomplete paraplegia. Emergency surgery for hematoma evacuation was performed, but no obvious hematoma was found. After the second surgical procedure, he showed paraplegic. When the patient was informed that a third operation was needed, he recovered almost completely without any treatment. This case reminds us that spine surgeons should be aware of possible conversion paralysis, especially in patients who develop a new neurological deficit after spinal surgery.

## Introduction

Conversion paralysis is a rare disease characterized by loss of sensory and motor functions; its most typical clinical feature is that the symptoms and neuroanatomy are inconsistent (1–3). While this condition has been observed for many centuries, its etiology remains unclear and is often associated with trauma or social events (4–9).

Cervical spondylotic myelopathy (CSM) is a common progressive disorder of the spinal cord (10, 11). Surgery, which is considered the best treatment, regularly stabilizes nerve function and may even lead to improvement in the patient's condition (4). Anterior cervical corpectomy decompression and fusion (ACCF) is often recommended for patients diagnosed with symptomatic CSM, but the major complications of spinal decompression may be unavoidable in some cases (5, 6). New neurological impairments caused by surgery must be promptly diagnosed and treated to reduce the risk of permanent neurological disability (7). However, paralysis after spinal surgery does not always originate from surgical complications.

Here, we report the case of a 67-year-old man who underwent an unnecessary emergency operation because of conversion paralysis after cervical surgery.

## Case Presentation

**Figure 1:**
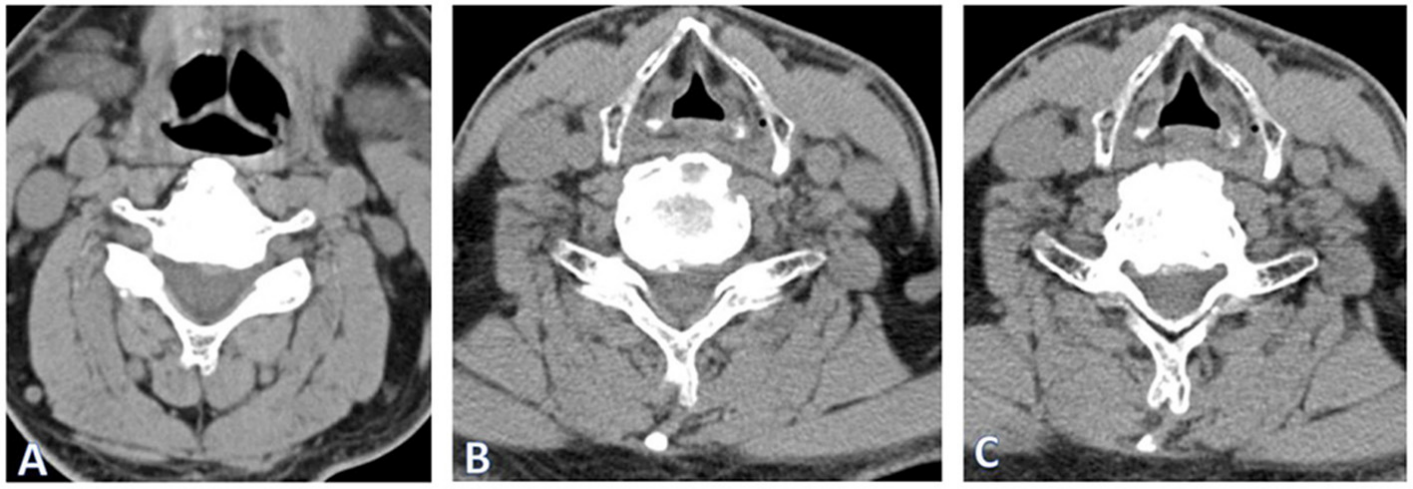
Cervical computed tomography (CT) showed that CT of the cervical spine showed spinal cord compression at the level of C 5/6 **(A)** and C 6/7 **(B)** and ossification of the posterior longitudinal ligament at the level of C 5–7 **(C)**.

A 67-year-old man living alone was admitted with unsteady gait due to CSM. He had received conservative treatment for more than 1 year, despite which his gait disturbance had progressed. Physical examination revealed normal muscle strength and tendon hyperreflexia in the lower extremities, but the Babinski sign was negative. The consultation of neurologists ruled out intracranial diseases. His medical history included hypertension and a bradycardia-related pacemaker, and he had no history of genetic diseases and mental disorders. Cervical computed tomography (CT) showed that the spinal cord was compressed by the protrusion of the C5/6 and C6/7 discs, and the ossification of the posterior longitudinal ligament from C5 to C7 (Figure 1).

ACCF was performed on the C6 vertebra, with the patient under general anesthesia. The whole process was successful, and the drainage tube was placed in the surgical site. The patient was not awake after the operation and went to ICU with endotracheal intubation. However, on awakening from the anesthesia, approximately 2 h after the operation, the patient presented with incomplete paraplegia. Physical examination revealed that the muscle tone of limbs was decreased, and the muscle strength of the upper and lower limbs were grade 3 and grade 2, respectively. There was no drainage from the surgical incision. Therefore, the genesis of incomplete paraplegia lurk in epidural hematoma.

**Figure 2:**
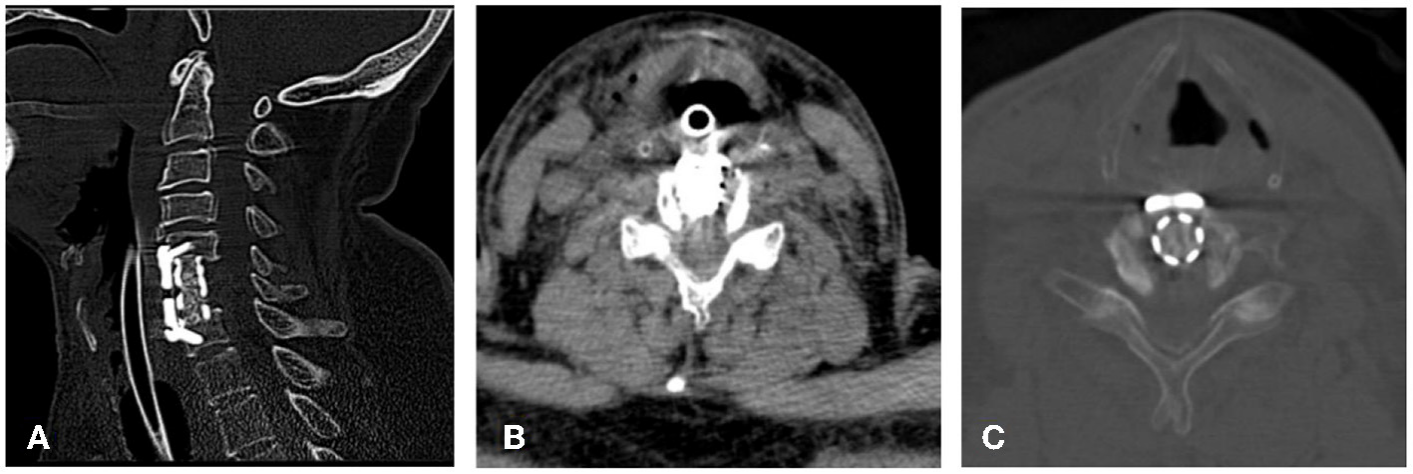
Emergency CT revealed that normal implant location **(A)** and spinal canal morphology without any occupational manifestations in the spinal canal **(B,C)**.

Emergency operation for hematoma evacuation was performed. There was no hematoma that would cause compression. Unfortunately, he showed paraplegia after waking from anesthesia. Physical examination at presentation demonstrated that the pain sensation disappeared below the nipple on both sides. The muscle strength of the upper and lower limbs was 2/5 and 0/5 grade, respectively, and the Babinski sign was positive on both sides. When high-dose methylprednisolone was used, the muscle strength of lower limbs is 1/5 grade. Emergency CT revealed that the implant was in position and the shape of the spinal canal was normal without any signs of an obvious space occupying lesion (Figure 2). When the patient was told a third operation may be needed, his sensory and motor function quickly recovered. The next day, the gait disturbance before operation completely disappeared. In psychiatrist's consultation and the postoperative psychological assessment, this condition was diagnosed as conversion paralysis. Later, his family told us that the patient had a similar experience in the past. His sensory and motor functions of upper limbs had disappeared after cardiac pacemaker implantation, but spontaneously completely recovered 2 days later. Meanwhile, his family said that they had cared for him during hospitalization. The patient was followed up for 3 years, roughly every 6 months. His motor and sensory conditions were completely normal in every follow-up.

## Discussion

CSM is a common progressive disorder of the spinal cord (10, 11). Surgical treatment is the best as it can stabilize and improve nerve function (4). ACCF is the one of recommended for patients diagnosed with symptomatic CSM (5, 6).

Conversion paralysis is a psychogenic non-organic loss of motor or sensory function, but the underlying cognitive, emotional, and neurophysiological mechanisms are unclear (1, 2, 8). It is always induced by some traumatic event; the prevalence of patients admitted to spinal wards with conversion disorder was 0.3–3.8% (1–3, 12–14). Although it is rare, conversion disorders after spinal trauma or spinal surgery have been reported occasionally (1, 7, 15, 16).

The clinical manifestations vary markedly among individuals, making diagnosis difficult. If a patient's symptoms are difficult to explain neuroanatomically or are functionally inconsistent, conversion paralysis should be considered as a differential diagnosis (17). However, it is very important to keep in mind that a diagnosis of conversion paralysis should never be made unless all possible organic causes have been ruled out (13). Many clinicians are reluctant to diagnose conversion disorder for fear of missing an organic cause for the patient's symptoms (1). Accurate diagnoses are imperative, as misdiagnosis may expose patients to unnecessary studies and treatments (2). Magnetic resonance imaging or CT can be used to find evidence of nervous system disorders, especially in postoperative patients, but defects obscured by implants can confuse examiners. Barre's sign was used to distinguish between organic and conversion paralysis, but only in patients with incomplete paralysis (18). The Spinal Injuries Center test is a new method for the diagnosis of conversion paralysis. This simple and non-invasive test allows for an accurate diagnosis of this disease (sensitivity, 100%; specificity, 97.9%) (13).

The prognosis of conversion disorder is extremely uncertain, ranging from complete recovery to none at all. Heruti et al. (2) reported that among 29 patients who underwent traumatic events and were diagnosed with conversion disorder, 8 recovered completely, 8 recovered partially, and 13 did not improve. Letonoff et al. (1) reported three cases of post-traumatic conversion disorder and reviewed the relevant literature; they suggested that complete recovery can be expected, often with rapid results.

To the best of our knowledge, only two cases of conversion disorder after cervical spine surgery have been reported in the past (15, 16). Boudissa et al. (16) reported a patient appeared left hemiplegia sparing of the face and complete motor loss, but sensations were normal after cervical spine arthroplasty of C6/7. The misdiagnosis of postoperative spinal epidural hematoma led to a second operation. Zhu et al. (15) reported that one patient had undergone anterior cervical decompression and fusion for disc herniation, leading to motor function loss of the extremities. The patient recovered immediately after placebo treatment. However, the clinical manifestations of both these cases were not consistent with the neuroanatomy of the surgical site.

In our case, the symptoms of paralysis after surgery were consistent with the spinal cord injury at the operation level. We misdiagnosed the postoperative spinal epidural hematoma and performed an exploratory operation. Psychiatrists believed that it was related to preoperative notification of complications and the patient's needs. First, he had a strong psychological demand for concern from his family. He subconsciously believed that if he was ill, his family would take care of him. Upper limb dysfunction after cardiac pacemaker implantation is that situation. Second, the doctor told the patient about the possible clinical symptoms if the operation failed. The information was reinforced in his subconscious. As a result, the ACCF showed symptoms that were consistent with the level of the operative spinal cord injury. This led clinicians to misjudge the postoperative spinal epidural hematoma after ACCF and led to unnecessary exploratory surgery.

In summary, conversion paralysis is a rare condition with various clinical manifestations, and surgical treatment is a common inducement. Spine surgeons should consider the possibility of conversion paralysis when patients have sensory and motor dysfunction after spinal operation. A detailed medical history inquiry is necessary. Although it is difficult to diagnose paralysis caused by conversion paralysis, especially after operation or trauma, accurate diagnosis is necessary because misdiagnosis may result in unnecessary treatment.

## Data Availability Statement

The original contributions presented in the study are included in the article/Supplementary Material, further inquiries can be directed to the corresponding author.

## Ethics Statement

Written informed consent was obtained from the patient for publication of this case report and any accompanying images.

## Author Contributions

All authors listed have made a substantial, direct, and intellectual contribution to the work and approved it for publication.

## Funding

This study was supported by grants from the Sichuan Provincial Science and Technology Key R&D Projects [No.2019YFS0282] and 1·3·5 project for disciplines of excellence-Clinical Research Incubation Project, West China Hospital, Sichuan University [No.2020HXFH049].

## Conflict of Interest

The authors declare that the research was conducted in the absence of any commercial or financial relationships that could be construed as a potential conflict of interest.

## Publisher's Note

All claims expressed in this article are solely those of the authors and do not necessarily represent those of their affiliated organizations, or those of the publisher, the editors and the reviewers. Any product that may be evaluated in this article, or claim that may be made by its manufacturer, is not guaranteed or endorsed by the publisher.
