# Supplementary material for: Conversion Paralysis After Cervical Surgery: A Case Report and Literature Review
Source: Front Surg. 2022 Feb 17;9:814498. doi: 10.3389/fsurg.2022.814498 (PMC8892200; doi:10.3389/fsurg.2022.814498)
Supplement: Supplementary file 1 [file Table_1.DOCX]

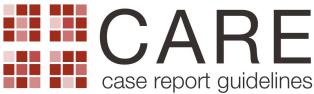
**CARE Checklist (2013) of information to include when writing a case report
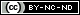
**

**Topic Item Checklist item description Reported on Page**

**Title 1** The words “case report” should be in the title along with the area of focus 1

**Key Words 2** 2 to 5 key words that identify areas covered in this case report 2

**Abstract 3a** Introduction—What is unique about this case? What does it add to the medical literature? 2-3

**3b** The main symptoms of the patient and the important clinical findings 3

**3c** The main diagnoses, therapeutics interventions, and outcomes 3-5

**3d** Conclusion—What are the main “take-away” lessons from this case? 8

**Introduction 4** One or two paragraphs summarizing why this case is unique with references 9-11

**Patient Information 5a** De-identified demographic information and other patient specific information 2

**5b** Main concerns and symptoms of the patient 2-4

**5c** Medical, family, and psychosocial history including relevant genetic information (also see timeline). 2-4

**5d** Relevant past interventions and their outcomes 2-4

**Clinical Findings 6** Describe the relevant physical examination (PE) and other significant clinical findings 2-4

**Timeline 7** Important information from the patient’s history organized as a timeline 2-4

# Diagnostic Assessment

**Therapeutic Intervention**

**Follow-up and Outcomes**

**8a** Diagnostic methods (such as PE, laboratory testing, imaging, surveys) 2-4

**8b** Diagnostic challenges (such as access, financial, or cultural) 2-4

**8c** Diagnostic reasoning including other diagnoses considered 2-4

**8d** Prognostic characteristics (such as staging in oncology) where applicable 4-5

**9a** Types of intervention (such as pharmacologic, surgical, preventive, self-care) 2-4

**9b** Administration of intervention (such as dosage, strength, duration) 2-4

**9c** Changes in intervention (with rationale) 2-4

**10a** Clinician and patient-assessed outcomes (when appropriate) 3-5

**10b** Important follow-up diagnostic and other test results 3-5

**10c** Intervention adherence and tolerability (How was this assessed?) 4

**10d** Adverse and unanticipated events 4

**Discussion 11a** Discussion of the strengths and limitations in your approach to this case 5-7

**11b** Discussion of the relevant medical literature 5-7

**11c** The rationale for conclusions (including assessment of possible causes) 5-7

**11d** The primary “take-away” lessons of this case report 5-7

**Patient Perspective 12** When appropriate the patient should share their perspective on the treatments they received 5-7

**Informed Consent 13** Did the patient give informed consent? Please provide if requested . . . . . . . . . . . . . . . . . . . . . . . . . . . . . . . . . . . . . . **Yes No**
